# Slowing of Parameterized Resting-State Electroencephalography After Mild Traumatic Brain Injury

**DOI:** 10.1089/neur.2024.0004

**Published:** 2024-04-18

**Authors:** Mark C. Nwakamma, Alexandra M. Stillman, Laurel J. Gabard-Durnam, James F. Cavanagh, Charles H. Hillman, Timothy P. Morris

**Affiliations:** ^1^ Department of Physical Therapy Human Movement Sciences, Northeastern University, Boston, Massachusetts, USA.; ^2^ Department of Psychology, Northeastern University, Boston, Massachusetts, USA.; ^3^ Center for Cognitive and Brain Health, Northeastern University, Boston, Massachusetts, USA.; ^4^ Department of Neurology, Beth Israel Deaconess Medical Center, Boston, Massachusetts, USA.; ^5^ Harvard Medical School, Boston, Massachusetts, USA.; ^6^ Department of Psychology, University of New Mexico, Albuquerque, New Mexico, USA.; ^7^ Department of Applied Psychology, Northeastern University, Boston, Massachusetts, USA.

**Keywords:** alpha peak frequency, aperiodic activity, EEG, mTBI, spectral parameterization

## Abstract

Reported changes in electroencephalography (EEG)-derived spectral power after mild traumatic brain injury (mTBI) remains inconsistent across existing literature. However, this may be a result of previous analyses depending solely on observing spectral power within traditional canonical frequency bands rather than accounting for the aperiodic activity within the collected neural signal. Therefore, the aim of this study was to test for differences in rhythmic and arrhythmic time series across the brain, and in the cognitively relevant frontoparietal (FP) network, and observe whether those differences were associated with cognitive recovery post-mTBI. Resting-state electroencephalography (rs-EEG) was collected from 88 participants (56 mTBI and 32 age- and sex-matched healthy controls) within 14 days of injury for the mTBI participants. A battery of executive function (EF) tests was collected at the first session with follow-up metrics collected approximately 2 and 4 months after the initial visit. After spectral parameterization, a significant between-group difference in aperiodic-adjusted alpha center peak frequency within the FP network was observed, where a slowing of alpha peak frequency was found in the mTBI group in comparison to the healthy controls. This slowing of week 2 (collected within 2 weeks of injury) aperiodic-adjusted alpha center peak frequency within the FP network was associated with increased EF over time (evaluated using executive composite scores) post-mTBI. These findings suggest alpha center peak frequency within the FP network as a candidate prognostic marker of EF recovery and may inform clinical rehabilitative methods post-mTBI.

## Introduction

Ninety percent of traumatic brain injury (TBI) cases presented to hospitals are mild in severity, in which approximately half of adult patients fail to return to pre-injury levels of health 6 months post-injury.^1^ Data from several large longitudinal cohort studies suggest mild traumatic brain injury (mTBI) to be a chronically evolving condition given that deficits in functional and behavioral outcomes have been reported up to 7 years post-injury.^2^ Cognitive impairment is a frequently reported symptom of mTBI.^3,4^ Studies have demonstrated persistent cognitive deficits post-mTBI even up to 12-months post-injury,^5^ which may lead to increased risk of neurodegenerative diseases such as dementia or Alzheimer's disease.^6^

Executive function (EF) is one domain of cognition that is typically affected by mTBI.^7,8^ Persons suffering from cognitive impairment show persistent deficits in subdomains of EF, such as working memory, cognitive/inhibitory control, and verbal fluency.^9–11^ Deficits in EF are directly related to damage to specific neural structures, particularly to regions within the frontoparietal (FP) control network.^12–14^ The FP network is a flexible, task-positive network whose nodes alter their connectivity with other network nodes.^15^ Altered connectivity between network nodes has been observed to be based on task objectives, facilitating learning novel tasks,^15^ and comprises functional connections within pre-frontal and parietal cortices.^16,17^ Neuropsychological assessments are typically used with the purpose of detecting changes in brain and cognitive outcomes post-injury to inform clinical treatment and prognosis,^18^ but are not sensitive enough to detect subclinical brain damage, which may better inform prognostic models of recovery. Therefore, studies have used various neuroimaging techniques to provide a more robust mechanistic understanding of the functional underpinnings of mTBI.^19,20^

Resting-state electroencephalography (rs-EEG) is a non-invasive, scalable neurophysiological technique that records fluctuations in brain electrical activity through synchronous post-synaptic potentials within the cerebral cortex in the absence of a task.^21^ Among other metrics, measuring changes in the brain's oscillatory electrical activity by EEG can provide a psychometrically robust measure of brain function. This EEG oscillatory activity is indexed commonly by relative and/or absolute spectral power within canonical frequency bands and have been correlated with cognition in various clinical populations.^22–25^ However, reported changes in power post-mTBI are conflicting and lack consensus. For example, several studies report an increase in power within slow-wave frequency bands post-mTBI.^26–28^ Conversely, other studies report an increase in power in higher frequency bands coupled with a decrease in lower frequency bands post-injury^29^ and/or a general decrease in both high- and low-frequency bands.^30,31^

Inconsistencies in past EEG-mTBI studies may be attributed to the dependency on determining spectral activity within pre-defined canonical frequency narrow bands (e.g., 8–12 Hz for the alpha band). This traditional approach can lead to a distortion and/or misrepresentation of the neural signal given that changes in spectral power may not reflect purely physiological oscillatory activity.^32,33^ Because EEG recordings are comprised of both oscillatory and aperiodic activity,^33^ average band power measures can not only be dominated by aperiodic neural activity instead of oscillatory, but may also reflect a broadband shift of the power spectrum across frequencies (for a review, see Donoghue and colleagues, 2020^32^). Parameterizing the neural signal into oscillatory and aperiodic components allows investigators to account for broadband shifts in power across frequencies (offset), the structure of aperiodic power across the spectrum (exponent), and detect peaks of oscillatory power above the aperiodic activity that cannot be isolated within traditional canonical power frequency bands.^32,34^ Thus, given the conflicting reports of changes in spectral power post-mTBI, parameterization of EEG scalp recordings across frequencies, though accounting for the aperiodic signal, may be an important analytical step to develop a greater understanding of the neuroplastic rhythmic and arrhythmic changes in brain activity post-mTBI.

Therefore, the purpose of this study was to determine differences in both rhythmic and arrhythmic activity in rs-EEG in those with a subacute (within 2 weeks of injury) mTBI versus those without. We conducted analyses across the whole brain as well as within the cognitively relevant FP region of interest (ROI), because our second aim was to test whether any changes in rhythmic or arrhythmic activity were associated with longitudinal changes in EF.

## Methods

### Study design

The data used in this article have been previously published in Cavanagh and colleagues (2019),^35^ Broadway and colleagues (2019),^10^ and Cavanagh and colleagues (2020)^36^ and are available on OpenNeuro (ds003522). The original study was conducted as a prospective cohort study design.

### Participants

Originally, participants were recruited from the Departments of Neurosurgery and Emergency Medicine from the University of New Mexico Health Science Center (UNMHSC) and persons within the Albuquerque community in Albuquerque, New Mexico. Eighty-eight participants (29.60 ± 10.92 years old, 37 females) provided written informed consent after the UNMHSC Human Protection Research Office approved the study protocol. mTBI patients were recruited within 14 days of injury, where they had a Glasgow Coma Scale of 13–15 coupled with loss of consciousness (≤30 min) post-injury. Injury history was assessed using a modified version of the Rivermead semistructured interview.^37^ The control group were sex and age matched. Neither mTBI nor control participants had a history of TBIs preceding participation in the study. Four participants with mTBI, along with 3 controls, were taking selective serotonin reuptake inhibitors. Exclusion criteria for the initial recruitment included: major medical or psychiatric conditions, ongoing or previous history of substance abuse, and could not speak fluent English. mTBI and control participants were invited to three testing sessions. The first session (held within 2 weeks of injury and subsequently referred to as “week 2”) was completed within 3–14 days post-injury and consisted of both EEG and cognitive testing. Cognitive testing was repeated approximately 2 and 4 months after the week 2 visit.

### Electroencephalography recording

Brain electrical activity was recorded using a 64-channel Brain Vision system EEG cap (LiveAmp 64). The vertical electrooculogram was recorded from bipolar auxiliary inputs. Electrical activity was measured across 0.5–100 Hz with a sampling rate of 500 Hz using CPz as an online reference and APz as ground. Eyes open and eyes closed (EC) rs-EEG data were extracted from the first and last 2 min of an auditory oddball task, and EC data were used for all subsequent analyses.

### Electroencephalography data processing

The raw rs-EEG data were pre-processed using HAPPE (version 3.3) within MATLAB 2020b software (The MathWorks, Inc., Natick, MA). HAPPE is a validated and standardized automated processing pipeline designed for EEG data containing high artifact and short recording length.^38^ EEG data were put through seven pre-processing steps: 1) All files were subject to a 1- to 100-Hz band-pass filter. 2) All channels in both cohorts were identified as the “EEG channel subset” and were selected for pre-processing. 3) The 60-Hz electrical line noise was removed from the data using cleanline multi-taper regression. 4) Identification of bad channels was determined using default multi-metric classification in HAPPE, such that bad channels were removed from data and interpolated in a later step. 5) Cleaned rs-EEG data were then subject to wavelet-based artifact correction using hard Bayesian thresholding to remove artifacts within the data throughout the spectrum. 6) Remaining rs-EEG data were segmented into 2-sec segments, and any segments with residual artifact were rejected using joint probability criteria. 7) Channels removed from step 4 underwent spherical interpolation of their signal, and then all channels were re-referenced to the average signal.

### Electroencephalography analysis: spectral parameterization

**FIG. 1.**
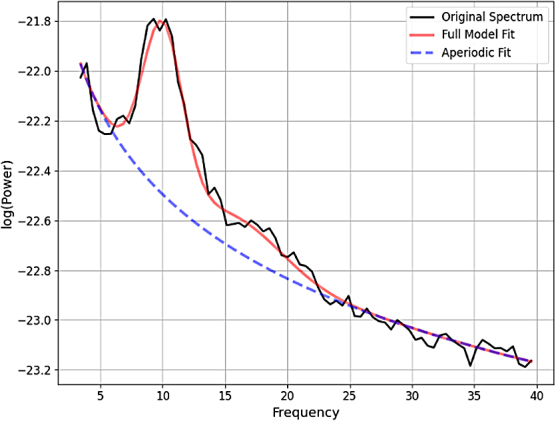
An example of a parameterized power spectrum in which the original spectrum, aperiodic fit (displaying simulated arrhythmic activity), and the full model fit (original spectrum accounting for aperiodic features) are presented.^33^

The rs-EEG power spectral densities (PSDs) were extracted from the EC EEG data using the Pwelch function (fast Fourier transform with a 2-sec Hamming window and 50% window overlap) in custom-written scripts within MATLAB 2020b (The MathWorks, Inc.). The transformed (non-corrected) PSDs were in 1- to 50-Hz frequency bins for each EEG channel. Individual whole-brain and FP-ROI power spectra were then extracted and decomposed into periodic and aperiodic spectral activity using the Fitting Oscillations One Over F (FOOOF)^32^ python toolbox (version 1.0.0) within the JupyterLab Notebook. Fz, F3, F4, Pz, P3, and P4 channels were selected to represent the FP-ROI because it has been used in previous EEG studies to represent the FP network.^39–41^ The FOOOF algorithm extracts both the aperiodic and periodic spectral activity from the extracted PSD using a model-based approach (Fig. 1).^32^ Aperiodic activity is characterized as a 1/f-like distribution that exponentially decreases in power across increasing frequencies and consists of the exponent and offset. The exponent exhibits the sequence of power across frequencies characterizing the steepness, or slope, of the diminishing power spectrum in the log-log space,^42^ whereas the offset is a parameter reflecting the scale-free uniform shift of power across frequencies.^32^

For the present study, the FOOOF settings were as followed: peak width limit (2–12), max number of peaks (8), minimum peak height (0), and peak threshold (2). These settings were applied to the algorithm, extracting both components, from a broad frequency range of 1–50 Hz (at a resolution of 1 Hz) to encompass all canonical periodic frequency bands of interest. Fitting was achieved using the “fixed” aperiodic mode. Parameterized periodic activity is reputed neural activity over and above the aperiodic component and consists of the center peak frequency, aperiodic-adjusted power, and bandwidth of any detected peaks. All participants had detectable peaks above the aperiodic component. Therefore, periodic metrics of detected peaks were extracted from each participant and used in the subsequent models. To capture any slowing or increase in dominant peaks resulting from brain injury, extended ranges of 5–15 (alpha) and 16–31 Hz (beta) were used to detect consistent peaks across participants.^43,44^

Further, the frequency and bandwidth corresponding with the highest aperiodic-adjusted power, within the extended ranges, were extracted from each participant and used in subsequent analyses. Together, outcome measures for parameterized rs-EEG using the FOOOF algorithm consisted of: center peak frequency of detected peaks, aperiodic-adjusted power at detected peaks, bandwidth of detected peaks, and the aperiodic metrics (exponent and offset).

A global goodness-of-fit metric (*R*^2^) was extracted to indicate whether the spectral parameterization model sufficiently fit the EEG data. Participants with *R*^2^ values <0.95 were characterized as having poor model fits and excluded from subsequent analyses (presented in Supplementary Fig. S1). To replicate previous work and add to the existing literature, we also tested relative spectral power within the pre-defined canonical frequency bands and compared those metrics between groups (Supplementary Material S2).

### Cognitive function testing

EF measures were collected from the NIH EXAMINER, which is a standardized neuropsychological battery used to assess distinct domains of EF.^45^ Several tasks were collected and grouped into three main subdomains of EF (cognitive control, working memory, and fluency) and used to calculate a final executive composite score as an accumulated performance metric of all tasks.

#### Domain: cognitive control

The *flanker* measures response inhibition by accuracy and reaction time per iteration. Participants were told to focus on a centered fixation point until a row of five arrows were presented above or below it. Once presented, the participants indicated whether the center arrow faced toward the left or right by pressing on the corresponding buttons. There were two conditions throughout the task. The congruent condition had the non-target arrows face the same direction as the center arrow, and the incongruent condition had the non-target arrows face the opposite direction of the center arrow. Stimuli were randomly presented with a variable duration of 1000–3000 ms per presentation. Software within the task generates a score from zero to ten and permits data to be combined.

The *continuous performance test* is an inhibitory task that instructs participants to respond to one type of stimulus and resist responding to others. Participants were instructed to respond as quickly and accurately as possible. Stimuli were presented focally on a computer screen and participants responded by pressing the left button for target images only. Non-target images were similar in size and shape to the target, of which participants attempted to not respond. The primary measure of the task was the total number of “false alarms” to the non-target images. A total of 100 experimental trials were presented, 20% of which were non-target images.

*Dimensional set shifting* is a task requiring participants to match the color or shape of a stimulus located on the top of the screen with one of two stimuli presented at the bottom corners of the screen. This task consists of two types of task blocks. In the task-homogeneous blocks, participants were instructed to match the top stimuli to one of the two bottom stimuli, by color or shape. In the task-heterogeneous blocks, participants were instructed to alternate randomly between color and shape. General and specific switch costs were measured by the integration of both homo- and heterogenous tasks. The two homogeneous tasks each presented 20 trials (20 color and 20 shape cue) whereas the heterogeneous tasks consisted of 64 total trials (32 color and 32 shape cue). Software within the task produces accuracy and reaction time and generates scoring, similar to the flanker task, by a scoring algorithm.

*The antisaccade task* measures controlled eye movements and consists of three blocks of trials where participants were instructed to focus on a focal fixation point on a screen.^46^ When a peripheral stimulus was presented, participants were instructed to move their eyes laterally either in the direction of the stimulus (prosaccade) or in the opposite direction (antisaccade), dependent on the block number. Targets were presented for 1000 ms.

#### Domain: working memory

*Dot counting* is a working memory task in which participants look at a mixed arrangement of green circles, blue circles, and blue squares on a computer screen. They are then instructed to count all the blue circles displayed on the screen and remember the total. Once the participant completed counting the blue circles, the administrator switched the display to a mixed arrangement of green circles, blue circles, and blue squares, after which the participant was instructed to count the blue circles in the new display. Displays presented in each trial increased (from two to seven) throughout the duration of the task. Once the participant counted all the blue circles presented in each display, they were asked to verbally recall the total number of blue circles in each of the different displays in the order presented. Participants were scored on the number of correctly recalled totals from each trial.

The spatial *n-back* task included both a spatial 1-back, consisting of updating one location at a time, and 2-back task, consisting of updating two locations at a time, to measure spatial working memory. During the task, participants were shown a display of white squares appearing in 15 different locations on a black computer screen, where each square was presented for 1000 ms. During the 1-back task, participants were instructed to press the left button whenever they saw the square located in the same location as the previous one and the right button if the square was located in a different location. After each response was given, the next square appeared on screen.

As the participants responded, a randomly selected number (between one and nine) appeared in the center of the screen ∼500 ms after each response was displayed for 1000 ms. Once the number was onscreen, the participant was required to verbally indicate the number before responding to the next square, thus preventing them from fixating on the location of the previously displayed square. The 1-back task was comprised of one block of 30 trials with 10 matching the previous squares' location and 20 not matching. The same instructions were given for the 2-back task; however, participants pressed the left, or right, button whenever a square was displayed in the same, or different, location of the previous square two trials back. The 2-back task encompassed one block of 90 trials (30 matching squares and 60 non-matching squares). Participants were instructed to respond as quickly and accurately as possible. The primary measure of the *n-back* performance was a d-prime measure representing the number of correct matches and non-matches.

#### Domain: fluency

*Phonemic fluency* is a paper-and-pencil task in which participants were instructed to name as many words as they could think of that began with a specific letter of the alphabet as quickly as they could. Two separate trials of phonemic fluency were given with 60 sec allotted for both trials. Participants were informed that numbers, nouns, and prefixes were not acceptable responses. The administrator of the task recorded all responses. Task performance for each letter was measured by the total number of correct responses, any violations to the rules, and repetitions of previously said words.

Similar to phonemic fluency, participants were instructed to produce as many objects belonging to a specific category as possible. This tasks also consisted of two separate categorical trials of 60 sec each. The same scoring was used to measure task performance, and all responses were recorded by the administrator.

Kramer and colleagues (2014)^45^ used a confirmatory factor analysis (CFA) to create a three-factor model with factors representing cognitive control, working memory, and fluency within the standardized battery. Results from the CFA analysis revealed the NIH EXAMINER can not only be defined by measures of cognitive control, working memory, and fluency, but can also be characterized by a global measure of EF. Item response theory was then used to create scores corresponding to all three metrics. Test-retest reliabilities ranged from 0.78 to 0.93. Participants within this cohort completed three separate sessions assessing EF at week 2, month 2, and month 4 post-injury.

### Statistical analysis

All statistical analysis was conducted using R software (Version 12.0; R Foundation for Statistical Computing, Vienna, Austria). Demographic data were compared between the mTBI and control groups with *t*-tests or a chi-squared test of proportions. Binomial logistic regression was used to test the relation between each cognitive test/rs-EEG power metric and mTBI diagnosis (mTBI vs. control) while controlling for years of education, age, and sex. Model assumptions were checked using *Q-Q* residual versus fitted plots to assume dichotomy of outcome variables and linearity of log odds. Log odds, representing the change in the log odds of the outcome variable given a 1-unit increase in the predictor variable, and ±95% confidence intervals (CI) are presented. *R*^2^ values from each model are displayed to illustrate the goodness-of-fit and how well the model explains the data variability. To test for an association between significantly different week 2 rs-EEG power metrics in the FP-ROI and EF in the mTBI group, linear mixed-effects models, with main effects of rs-EEG power metric, time (week 2, month 2, and month 4), and a rs-EEG*time interaction coupled with a participant-specific random slope and intercept were performed separately for each EF outcome.

False discovery rate (FDR)-corrected simple slopes were tested *post hoc* to determine any marginal effects of the interaction term. FDR-corrected *p* values are presented. Individual multiple linear regression models were conducted to determine any associations between significant week 2 rs-EEG power metrics, within whole-brain and FP electrodes, and EF tasks at all time points. Regression model assumptions were checked using the previously mentioned methods, in addition to a non-constant variance test and Shapiro-Wilk test for normality of residuals. *R*^2^ values from each model are displayed to illustrate the goodness-of-fit and how well the model explains the data variability. Significant influential outliers were checked in all models using Cooke's distance with a cutoff of 0.5. Years of education, age, and sex were accounted for in all models.

## Results

### Participant characteristics

Eighty-eight adults (37 female; 29.60 ± 10.92 years of age; range, 18–55) participated. There was a significant difference in years of education between groups, and given that participants were age and sex matched, no significant differences were observed in age or sex (Table 1).

**Table 1.** Demographics and Descriptive Statistics

***Participants' characteristics***				
	***mTBI***	***Control***	*p* ***value***	***CI***
*N*	56	32		
Age (mean SD)	29.25 (10.82)	30.21 (11.25)	0.695	–5.885 to 3.947
Sex: female (%)	22 (39)	15 (47)	0.638	–0.315 to 0.163
Days until first visit (mean SD)	10.42 (3.26)	NA		
***Years of education (mean SD)***	***13.48 (2.16)***	***14.62 (2.76)***	0.049^*^	***–2.283 to −0.002***

mTBI, mild traumatic brain injury; CI, confidence interval; SD, standard deviation; ^*^p < 0.05.

### Differences in aperiodic activity from parameterized resting-state electroencephalography

Logistic regression models revealed no significant between-group differences of aperiodic activity in the week 2 whole-brain aperiodic offset (log(odds) = −0.832, *p* = 0.421, CI = −2.928 to 1.168) or exponent (log(odds) = −0.926, *p* = 0.4374, CI = −3.307 to 1.423). Similarly, no significant between-group differences in either week 2 aperiodic offset (log(odds) = −1.045, *p* = 0.212, CI = −2.763 to 0.553) or exponent (log(odds) = −1.026, *p* = 0.362, CI = −3.297 to 1.166) were observed within the FP-ROI.

### Differences in periodic activity from parameterized resting-state electroencephalography

**FIG. 2.**
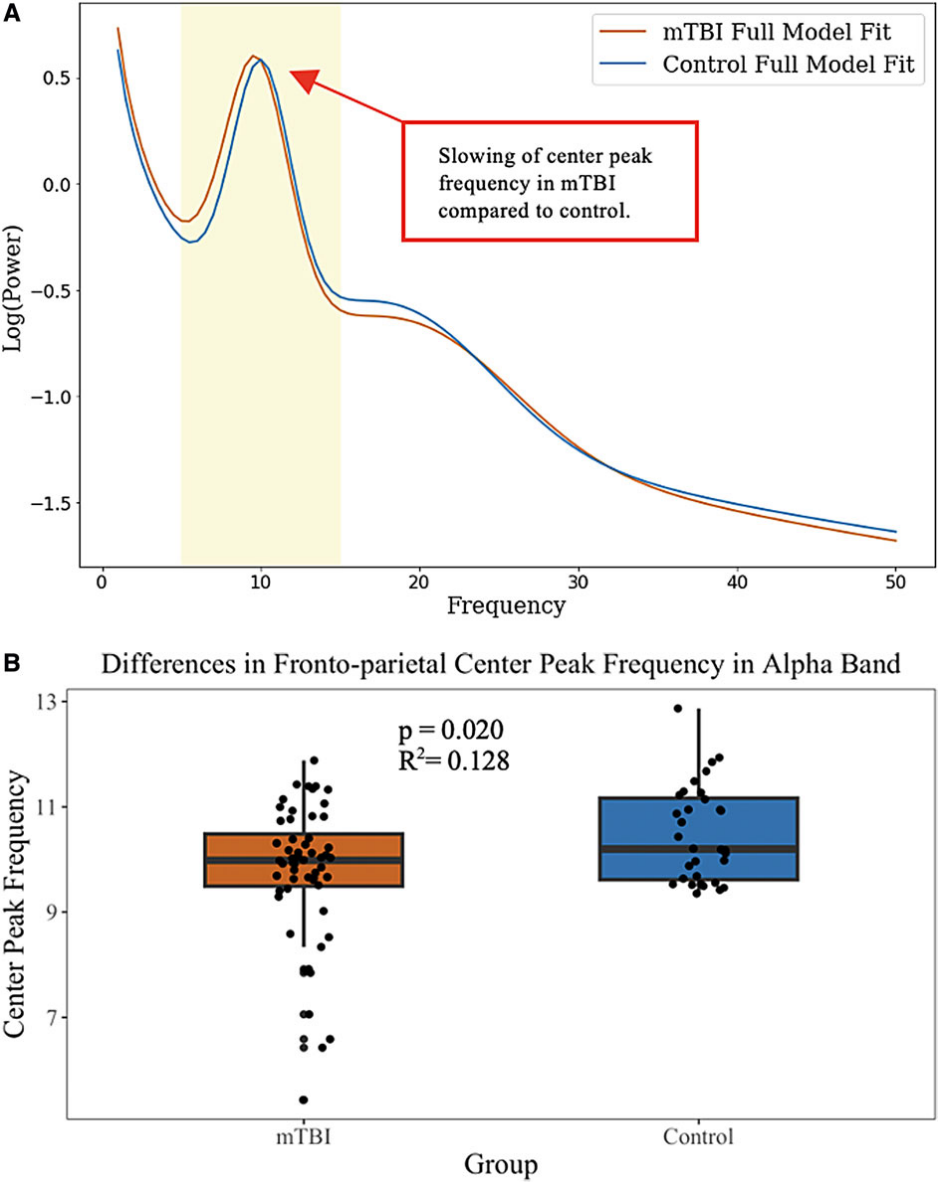
(**A**) Full model fit spectra, accounting for the aperiodic component, displaying differences in group mean alpha center peak frequency within the 5- to 15-Hz extended alpha range (shaded). (**B**) Box plots illustrating differences in mean alpha center peak frequency between mTBI and control groups. Center peak frequency is in units of hertz. mTBI, mild traumatic brain injury.

Whole-brain spectral parameterization detected a mean number of 3.94 ± 1.57 peaks in the mTBI group across the whole broadband frequency range (1–50 Hz) in the whole-brain electrode array. In the control group, a mean number of 3.03 ± 1.23 peaks were detected. At the group level, two prominent peaks across the entire broadband frequency were observed. The group-averaged first peak is considered to fall within the canonical alpha range (8–12 Hz) and the second within the canonical beta1 range (13–21 Hz). No significant between-group differences of periodic activity in week 2 whole-brain center peak frequency, aperiodic-adjusted power, or bandwidth at detected peaks within the alpha or beta1 range were found (Table 2). In the FP-ROI, a mean number of 4.26 ± 1.43 peaks were detected in the mTBI group and 4.37 ± 1.31 peaks were detected in the control. Logistic regression models revealed a significant between-group difference in periodic activity in week 2 center peak frequency in the alpha band within the FP electrodes (Fig. 2). No significant differences in aperiodic-adjusted power or bandwidth were observed in the alpha peak nor in any periodic metrics within the beta1 peak (Table 2).

**Table 2.** Logistic Regression Models, Coupled With Mean and Standard Deviation, for Between-Group Whole-Brain and FP Periodic Activity Differences With Significant Effects

***Week 2 periodic activity from parameterized rs-EEG***							
			***mTBI***	***Control***	***Log(odds)***	***p value***	***CI***
Whole brain	Alpha	Center peak frequency	9.898 ± 1.106	10.272 ± 0.891	0.407	0.094	–0.049 to 0.916
		Power	0.977 ± 0.432	1.029 ± 0.350	0.555	0.374	–0.654 to 1.822
		Bandwidth	1.404 ± 0.477	1.558 ± 0.471	0.714	0.140	–0.242 to 1.684
	Beta	Center peak frequency	19.603 ± 3.247	19.540 ± 2.618	0.042	0.594	–0.118 to 0.201
		Power	0.377 ± 0.176	0.440 ± 0.182	–3.277	0.204	–1.048 to 4.384
		Bandwidth	3.045 ± 1.737	3.664 ± 1.479	–1.814	0.869	–0.060 to 0.501
Frontoparietal	Alpha	***Center peak frequency***	***9.760 ± 1.311***	***10.476 ± 0.913***	***0.592***	0.020^*^	***0.131 to 1.136***
		Power	1.119 ± 0.445	1.131 ± 0.333	–0.264	0.662	–1.471 to 0.931
		Bandwidth	1.430 ± 0.525	1.399 ± 0.516	–0.238	0.607	–1.198 to 0.647
	Beta	Center peak frequency	20.459 ± 2.891	19.961 ± 2.696	–0.051	0.546	–0.225 to 0.113
		Power	0.472 ± 0.196	1.131 ± 0.208	–2.876	0.204	–0.790 to 3.824
		Bandwidth	3.560 ± 1.647	3.587 ± 1.532	–0.061	0.674	–0.351 to 0.224

FP, frontoparietal; rs-EEG, resting-state electroencephalography; mTBI, mild traumatic brain injury; CI, confidence interval; ^*^p < 0.05.

### Differences in cognition

**FIG. 3.**
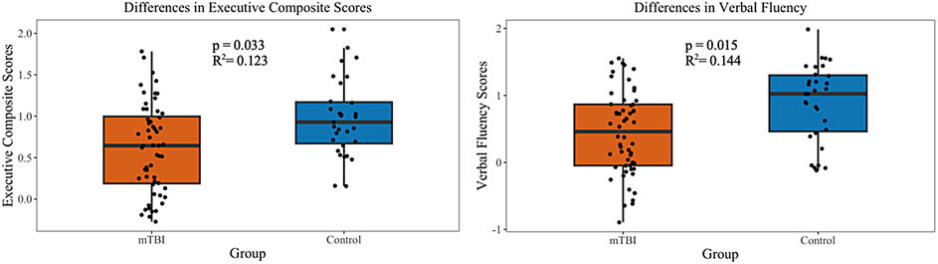
Box plots illustrating between-group differences in mean week 2 executive composite scores (left) and verbal fluency (right).

Significant differences in week 2 verbal fluency subscore (log(odds) = 1.060, *p* = 0.015, CI = 0.233–1.964) and the EF composite scores (log(odds) = 1.140, *p* = 0.033, CI = 0.131–2.253) were observed, where the mTBI group scored lower in both assessments in comparison to the control group (Fig. 3). No significant differences were observed in week 2 cognitive control or working memory subscores (Table 3).

**Table 3.** Logistic Regression Model Outcomes, Coupled With Mean and Standard Deviations, for Between-Group Differences in Executive Function Metrics With Significant Effects

***Group differences in week 2 executive function***					
	***mTBI***	***Control***	***log(odds)***	*p* ***value***	***CI***
Cognitive control	0.803 ± 0.660	1.016 ± 0.453	0.452	0.308	−0.410 to 1.349
***Fluency***	***0.440 ± 0.620***	***0.893 ± 0.578***	***1.060***	0.015^*^	***0.233–1.964***
Working memory	0.317 ± 0.634	0.496 ± 0.708	0.334	0.371	−0.389 to 1.090
***Executive composite score***	***0.614 ± 0.537***	***0.974 ± 0.461***	***1.140***	0.033^*^	***0.131–2.253***

mTBI, mild traumatic brain injury; CI, confidence interval; ^*^p < 0.05.

### Week 2 resting-state electroencephalography metrics correlate with changes in cognition over time

**FIG. 4.**
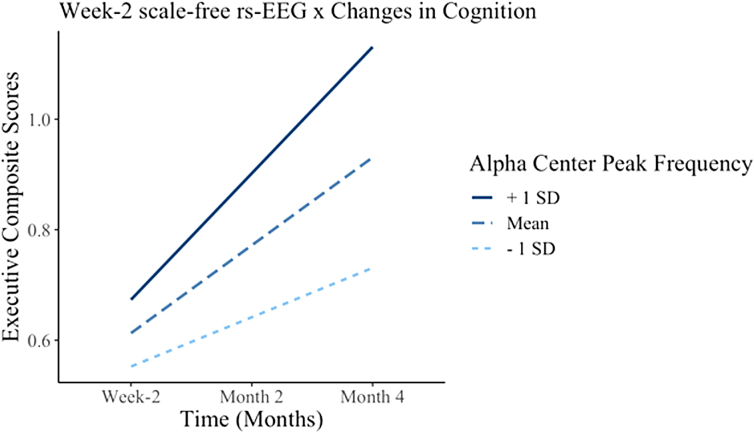
Interaction plot illustrating the relationship between executive composite scores and the time*alpha center peak frequency interaction term. Dashed lines represent the mean, the +1 SD is represented by the solid lines, and the −1 SD is represented by the dotted lines. EEG, electroencephalography; SD, standard deviation.

In response to the significant between-group differences within EF metrics and alpha center peak frequency in the FP-ROI, we tested whether changes in EF over 4 months were a function of changes in week 2 alpha center peak frequency in the mTBI group: linear mixed-effects models with week 2 rs-EEG power metrics; time (week 2, month 2, and month 4); an rs-EEG power metric by time interaction as fixed effects; and participant-specific random effects. A significant time*alpha center peak frequency (β = 0.049, *p* = 0.039, CI = 0.002–0.096, *Conditional R^2^* = 0.777) interaction was observed. *Post hoc* analyses, using FDR-corrected marginal effects, taking alpha center peak frequency at its mean, and 1 standard deviation (SD) above the mean, revealed that alpha center peak frequency at its mean (β = 0.12, *p* = 0.02) and +1 SD from the mean (β = 0.20, *p* = 0.01) were associated with increased executive composite scores over time, whereas −1 SD below the mean (β = 0.10, *p* = 0.06) was not significantly associated with increased composite scores over time (Fig. 4). We did not find a significant time*alpha center peak frequency interaction with verbal fluency (β = 0.045, *p* = 0.463, CI = −0.077 to 0.168, *Conditional R*^2^ = 0.654).

## Discussion

We found that after parameterizing the rs-EEG signal into rhythmic and arrhythmic oscillatory components, a significant slowing of center peak frequency within the alpha band was revealed in participants with subacute mTBI within a cognitively relevant FP network. Further, individual differences in alpha center peak frequency were associated with EF over 4 months post-injury, showing that those with alpha center peak frequency at or above the mean (closer to the healthy control group) were associated with significantly improved EF over time.

When parameterizing the rs-EEG signal to account for the aperiodic component, and to detect peaks across broadband frequencies, we observed only alpha center peak frequency, not power or bandwidth at center peak frequency or aperiodic metrics (exponent and offset), differing in those with a subacute mTBI compared to age- and sex-matched controls. However, when attempting to replicate previous mTBI studies quantifying power within *a priori* canonical frequency narrow bands (Supplementary Table S2), we found an increase in spectral power within theta coupled with a decrease in spectral power within beta1. These findings are consistent with some past literature,^47,48^ yet inconsistent with other past literature that reported decreases in slow- and/or high-wave-frequency power.^28–31^ Consequently, because of a lack of between-group differences in power metrics when accounting for the aperiodic component across broadband frequencies, it is plausible that discrepancies regarding changes in spectral power post-mTBI may be on account of excluding the aperiodic signal from analyses. Despite there being no observed significant between-group differences in the aperiodic exponent or offset, we can still assume there to be underlying aperiodic activity affecting spectral power post-mTBI.

Donoghue and colleagues (2020)^32^ demonstrated that oscillatory power is influenced by age-related changes in the aperiodic component, given that it led to a shift in narrowband alpha power unrelated to the spectral power within the narrowband preceding aperiodic inclusion. As such, power differences were not solely driven by periodic activity, but that the magnitude in spectral power was influenced by accompanying changes in aperiodic activity.^32^ Therefore, the disappearing between-group differences we observed, displaying a shift in spectral power from high to low frequencies within traditional canonical frequency bands, may be the result of power differences appearing less drastic after accounting for changes in the aperiodic component. Our results illustrate the apparent need for investigators to observe spectral changes across broadband frequencies and not solely within pre-defined frequency bands. In addition, pre-defined frequency bands minimize the ability to account for interindividualized variability in oscillatory activity, especially after injury. This is apparent within mTBI given that several participants had a dramatic slowing in alpha center peak frequency observed outside of the traditional 8- to 12-Hz frequency band, which would not have been captured if the neural signal was not parameterized across frequencies.

Slowing of alpha center peak frequency, accounting for the aperiodic component, was more pronounced than the slowing of canonical alpha peak frequency. This difference was observed within the FP network ROI, but not the whole brain. Slowing of alpha peak frequency, resulting from mTBI, has been reported in several EEG-mTBI studies, which we replicate in this study.^49,50^ Similar results are also observed in stroke, hippocampal atrophy, and Alzheimer's disease,^51–53^ indicating slowing of alpha peak frequency as a potential biomarker of general brain injury. However, current studies only report shifts in alpha peak frequency in canonical frequency bands and do not consider the aperiodic component in analyses. We demonstrate the importance of parameterizing the neural signal in this study given that the between-group differences in whole-brain alpha peak frequency disappear after accounting for the aperiodic component. To that end, existing evidence reporting changes in alpha peak frequency may be misrepresented or distorted by only relying on periodic changes in mean spectral power. Interestingly, in our results, the between-group differences of center peak frequency within the alpha band were only observed in the FP network. It remains unclear what mechanisms are at play resulting in a slowing of alpha center peak frequency specifically within this intrinsic task-positive network.

Diffuse axonal injury is a hallmark sign of TBI.^54^ Both long- and short-range connections within and between neural networks are susceptible to damage post-mTBI.^55^ This is attributable to the shear and tensile forces placed upon cortical axons at the time of injury and the secondary injury mechanisms initiated early after injury.^56^ A majority of reported impacts to the head occur frontally, and as such, those with mTBI are potentially at greater risk of frontal lobe dysfunction.^57^

Several studies report alpha oscillations being generated by complex interactions of thalamic and alpha pacemaker units within frontal regions and by corticocortical, corticothalamic, and thalamocortical connectivity propagating alpha waves after an anterior to posterior gradient.^58–62^ Because changes in white matter structures have been associated with alpha oscillations, which are sensitive to thalamocortical connections,^63,64^ our results suggest that a slowing of alpha center peak frequency within the FP network may be related to damaged cortical alpha structures within the FP network. We found that between-group differences in alpha center peak frequency within the FP network were driven primarily by differences in the frontal electrodes (Supplementary Fig. S3). To that end, slowing of alpha center peak frequency post-mTBI may be generated by damaged frontal cortical alpha pacemakers attempting to propagate alpha oscillations along injured axons primarily within the FP network.

Further, we provide additional evidence that brain changes post-mTBI are directly linked to cognitive deficits, showing that reduced alpha center peak frequency within the FP network predicts longitudinal improvements in EF. Past evidence has shown that whole-brain canonical alpha peak frequency is associated with verbal abilities,^65^ working memory,^66^ visual perception,^67^ and general intelligence^68^ in healthy and impaired populations. However, regarding alpha oscillations within the FP network, few studies report exclusively on alpha peak frequency, but rather on alpha phase synchrony, frequency coupling, and/or modulation of alpha rhythm.^69–72^

Of the few studies, Bertaccini and colleagues (2022)^73^ reported faster right parietal alpha peak frequency being associated with improved performance in a visuospatial working memory task in a cohort of 25 healthy adults. In addition, another previous rs-EEG study reported a strong significant correlation between higher individual alpha frequency and increased cognitive performance in both healthy younger and older adults.^68^ As such, the impairment in executive function, resulting from TBI exposure disrupting cortical structures within the FP network, may be a function of injury-induced slowing of alpha center peak frequency within the FP network.^12,74,75^ Such findings suggest alpha center peak frequency within the FP network as a potential prognostic marker of EF recovery post-mTBI.

Future research should look to expand the findings regarding region-specific associations of aperiodic-adjusted center peak frequency and longitudinal cognitive performance in persons with mTBI. Nevertheless, our results should be interpreted considering several limitations. The initial analysis is cross-sectional, with no pre-injury data available, despite pre-injury status likely considerably affecting the speed of recovery of those with mTBI.^76^ Our analysis only focused on EF, which though being particularly vulnerable to impairment post-mTBI,^10^ is not the only cognitive domain affected by mTBI. Additionally, a significant dropoff was observed regarding the collection of EF metrics at month 4 (see the Supplementary Material). However, the use of linear mixed-effects models implicitly accounts for missing data at random.

## Conclusion

The observed between-group differences in aperiodic-adjusted alpha center peak frequency within the FP network, coupled with the association with longitudinal changes in cognition, offers a parsimoniously novel mechanism of brain injury and cognitive recovery post-injury. Inclusion of the aperiodic component is not only potentially clinically relevant, regarding cognitive recovery, but also appears vital for a comprehensive understanding of changes in brain function post-mTBI.

## Acknowledgments

This work was previously presented in an abbreviated form on a poster at the 2023 Society for Psychophysiology Research Annual Meeting. We thank Alexa Monachino and Martina Kopčanová for their contributions to the methods and software tools used for this report.

## Authors' Contributions

Mark C. Nwakamma: methodology, software, formal analysis, investigation, writing–original draft, visualization, conceptualization. Alexandra M. Stillman: writing–review & editing. Laurel J. Gabard-Durnam: resources, methodology, writing–review & editing. James F. Cavanagh: funding acquisition, data curation, writing–review & editing. Charles H. Hillman: supervision, writing–review & editing. Timothy P. Morris: administration, conceptualization, supervision, writing–review & editing, methodology, formal analysis.

## Funding Information

This research was supported by an Institutional Development Award (IDeA) from the National Institute of General Medical Sciences of the National Institutes of Health under grant number P20GM109089 awarded to James F. Cavanagh.

## Author Disclosure Statement

No competing financial interests exist.

## Supplementary Material

Supplementary Table S1

Supplementary Figure S1

Supplementary Material S2

Supplementary Figure S2

Supplementary Table S2

Supplementary Figure S3

## Supplementary Material

Supplemental data

## Abbreviations Used

CFA: confirmatory factor analysis
CI: confidence interval
EC: eyes closed
EEG: electroencephalography
EF: executive function
FDR: false discovery rate
FOOOF: Fitting Oscillations One Over F
FP: frontoparietal
mTBI: mild traumatic brain injury
PSDs: power spectral densities
ROI: region of interest
rs-EEG: resting-state electroencephalography
SD: standard deviation
TBI: traumatic brain injury
UNMHSC: University of New Mexico Health Science Center
