# Supplementary material for: Slowing of Parameterized Resting-State Electroencephalography After Mild Traumatic Brain Injury
Source: Neurotrauma Rep. 2024 Apr 18;5(1):448–61. doi: 10.1089/neur.2024.0004 (PMC11044859; doi:10.1089/neur.2024.0004)
Supplement: Supplemental data [file Suppl_Data.pdf]

SUPPLEMENTARY

**Supplementary table 1**	Differences in EEG preprocessing quality control			
	mTBI	Control	p-value	CI
File Length (seconds)	120.12 ± 5.19	116.81 ± 15.01	0.235	-2.25 - 8.87
# of good channels selected	60.92 ± 2.13	59.81 ± 4.01	0.152	-0.42 - 2.65
% of good channels selected	95.20 ± 3.34	93.45 ± 6.26	0.152	-0.66 - 4.15

Supplementary table 1: Descriptive statistics of rs-EEG preprocessing quality control between mTBI and control individuals.

1. FOOOF goodness-of-fit metrics

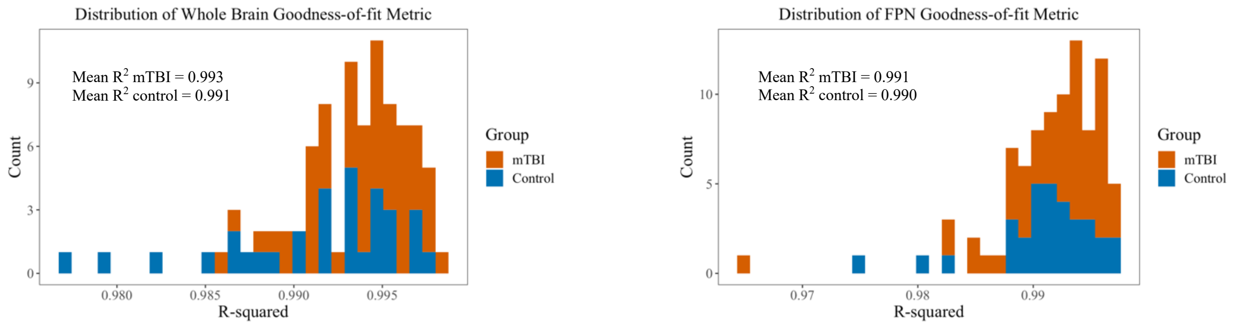

Supplementary figure 1: Histograms displaying distributions of goodness-of-fit metrics from whole brain and FP network FOOOF models. Mean goodness-of-fit metrics, in either whole brain or FPN FOOOF models, are above the 99th percentile.

2. Differences in relative power within canonical frequency bands

The canonical frequency bands were defined as delta (1- 4Hz), theta (5 – 8Hz), alpha (9 – 12Hz), beta1 (13 – 21Hz), beta2 (22 – 30Hz), and gamma (31-50Hz). Logistic regression models revealed a significant difference in whole brain week-2 relative theta power where an increase in power was detected in the mTBI group in comparison to the control group **SuppFig.2**. In addition, a significant difference in FP week-2 relative theta power and beta1 power, where an increase in theta, coupled with a decrease in beta1, was detected in the mTBI group in comparison to the control group (Table 2). However, we would observe significant between-group differences in week-2 alpha peak frequency across the whole brain and within FP electrodes. A reduction, or slowing, of alpha peak was detected in the mTBI group in comparison to the control group.

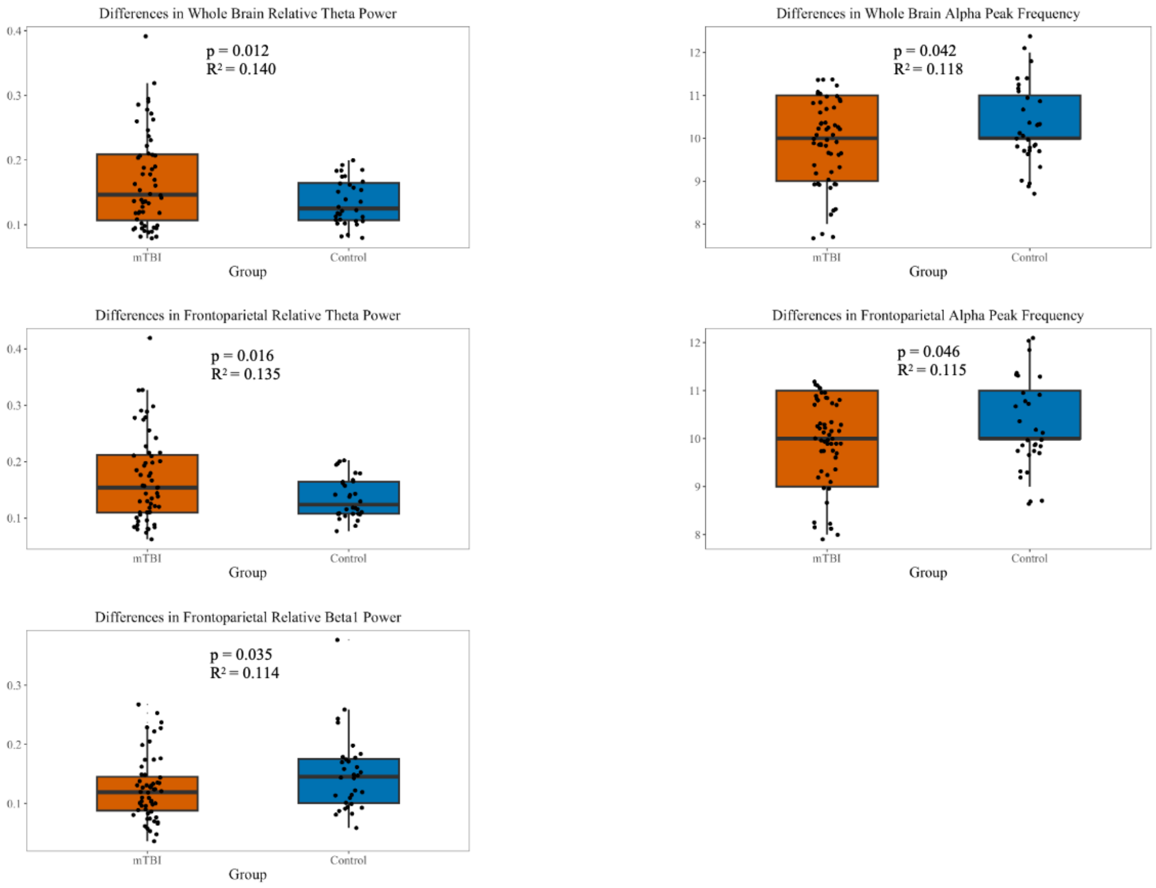

Supplementary figure 2: Box plots illustrating differences in mean canonical relative power in whole brain and frontoparietal electrodes between mTBI (burnt orange) and control (blue) groups.

Table 2 week-2 rs-EEG relative power

		mTBI	Control	Log(odds)	p	CI
Whole Brain	Delta	0.248 ± 0.078	0.243 ± 0.090	-1.388	0.612	-6.897 - 3.958
	Theta	***0.168 ± 0.072***	***0.135 ± 0.035***	***-12.104***	***0.012****	***-22.453 - -3.249***
	Alpha	0.341 ± 0.162	0.348 ± 0.144	0.653	0.675	-2.438 - 3.746
	Beta1	0.118 ± 0.048	0.133 ± 0.054	6.817	0.157	-2.519 - 16.604
	Beta2	0.047 ± 0.030	0.044 ± 0.026	-3.277	0.71	-21.733 - 13.598
	Gamma	0.040 ± 0.024	0.038 ± 0.021	-1.814	0.869	-24.307 - 19.535
	Alpha Peak Frequency	***9.875 ± 0.954***	***10.281 ± 0.851***	***0.556***	***0.041***	***0.042 - 1.124***
Frontopariet al	Delta	0.225 ± 0.082	0.217 ± 0.097	-1.92	0.487	-7.545 - 3.415
	Theta	***0.170 ± 0.078***	***0.135 ± 0.036***	***-10.635***	***0.016****	***-20.096 - -2.533***
	Alpha	0.390 ± 0.177	0.402 ± 0.154	0.833	0.566	-2.010 - 3.745
	Beta1	***0.124 ± 0.054***	***0.149 ± 0.064***	***9.183***	***0.035****	***0.871 - 18.238***

Beta2	0.052 ± 0.041	0.048 ± 0.030	-2.876	0.674	-17.458 - 10.043
Gamma	0.032 ± 0.024	0.030 ± 0.018	-3.074	0.794	-28.156 - 19.333
Alpha Peak Frequency	***9.892 ± 0.966***	***10.312 ± 0.895***	***0.529***	***0.046****	***0.029 - 1.082***

Table 2: Logistic regression models, coupled with mean and standard deviation, for between-group whole brain and frontoparietal canonical frequency power band differences with significant effects.

3. ROI-based differences in rs-EEG metrics within FP network

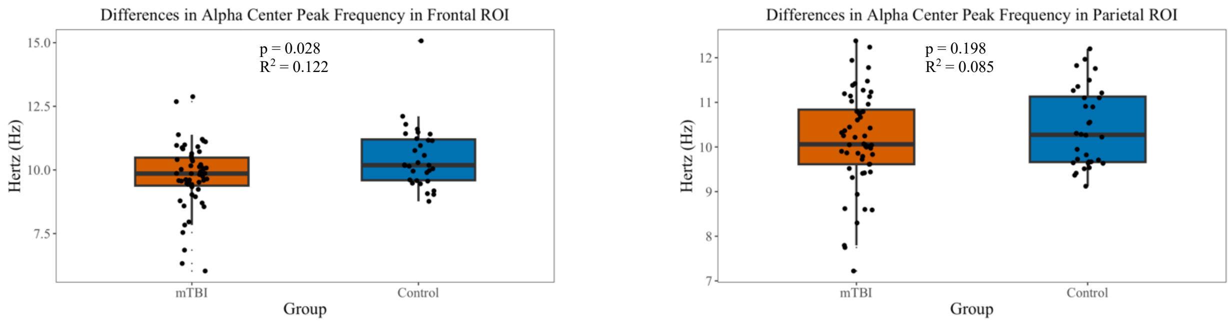

Supplementary figure 3: Boxplots displaying differences in center peak frequency within the alpha band between frontal and parietal electrodes. The between-group significant difference in FP center peak frequency within the alpha band may be driven by the frontal ROI within this cohort.
